# Improved Sealed Venous Indwelling Needle in Ultrasound‐Guided Thyroid Cyst Puncture Sclerotherapy: Characteristics and Applications

**DOI:** 10.1155/rrp/5868827

**Published:** 2026-07-31

**Authors:** Qing Zhang, Guihua Wu, Guoyan Zhu

**Affiliations:** ^1^ Department of Ultrasound, Wuzhong People’s Hospital, Suzhou, Jiangsu, China

**Keywords:** sealed venous indwelling needle, thyroid cyst, ultrasound-guided sclerotherapy, ultrasound intervention

## Abstract

**Objective:**

To assess the safety and efficacy of an improved sealed venous indwelling needle for ultrasound‐guided sclerotherapy of thyroid cysts in comparison with the standard percutaneous transhepatic cholangiography (PTC) needles.

**Methods:**

Two trapezoidal incisions (2 mm in topline, 1 mm in baseline, and 1 mm in height) were made along the needle core groove at the soft distal end of a sealed venous indwelling needle to create a novel open‐window sealed venous indwelling needle. To evaluate the efficacy and safety of this improved needle, we conducted a prospective cohort study with a historical control group, comparing the improved needle with standard 21‐gauge PTC needles in the treatment of 68 cystic thyroid nodules. Key outcomes included technical success, which encompasses successful needle placement, complete aspiration, and sclerosant delivery, as well as a reduction in cyst volume at the 3‐month follow‐up, and procedural outcomes, complications (bleeding, infection, and extravasation), procedural pain, and patient satisfaction (assessed using a 5‐point Likert scale).

**Results:**

Sixty‐eight cysts in 52 patients (median age, 54 years) were treated. Technical success was achieved in 94.1% (32/34) procedures versus 87.5% (28/34) in the PTC needle cohort (*p* = 0.27). The mean cyst volume reduction was 82.51% (range, 52%–100%) at 3 months. Complications occurred in 3 (two hematomas and one transient hyperthyroidism), with no infections or nerve injury. Patient‐reported pain scores were 1 (IQR 1‐2) and dropped by 43%. Overall satisfaction was 97% (66/68). The improved sealed venous indwelling needle was significantly superior to the PTC needle in terms of procedural pain, comfort, complication rate, and overall patient experience (*p* < 0.05), whereas no statistically significant differences were observed in recovery speed, involution efficacy, operator skill, or communication clarity (*p* ≥ 0.05).

**Conclusion:**

The improved sealed venous indwelling needle provides higher technical success, greater cyst volume reduction, fewer complications, and less pain than PTC needles, offering a safer and more effective alternative for ultrasound‐guided thyroid cyst sclerotherapy.

## 1. Introduction

Thyroid cysts, which are fluid‐filled cavities within the thyroid gland, have been a subject of medical interest for decades because of their prevalence and potential impact on patient health [1, 2]. Initially, the management of these cysts was primarily observational, with intervention considered only when symptoms such as neck discomfort, dysphagia, or cosmetic concerns developed. Traditional treatment methods, including aspiration and surgical removal, have been the mainstay of therapy for many years. While thyroid nodule aspiration is a minimally invasive procedure, it is associated with a significant risk of recurrence, which can lead to the need for additional interventions and may elevate patient discomfort and anxiety. Surgical interventions, while providing a more definitive solution, are not without their risks. These include infection, bleeding, and the potential for hypothyroidism due to damage to the thyroid gland. However, with an experienced surgical team and proper postoperative management, the probability of these complications can be significantly reduced. Ultrasound‐guided sclerotherapy using ethanol or lauromacrogol has demonstrated a reduction in recurrence rates. Considering that sharp percutaneous transhepatic cholangiography (PTC) needle tips can cause pain and bleeding, and that minor perforations may complicate the suction of cystic fluid [3–5], we have developed an improved sealed venous indwelling needle characterized by multiple through windows, a soft texture, and a blunt tip design. This prospective cohort study with a historical control group aims to evaluate the technical success rate, the 3‐month cyst volume reduction rate, the incidence of complications, operational pain, and patient satisfaction associated with ultrasound‐guided sclerotherapy using the improved needles, in comparison to outcomes obtained with PTC needles.

## 2. Materials and Methods

### 2.1. Development of an Open‐Window Sealed Venous Indwelling Needle

**FIGURE 1:**
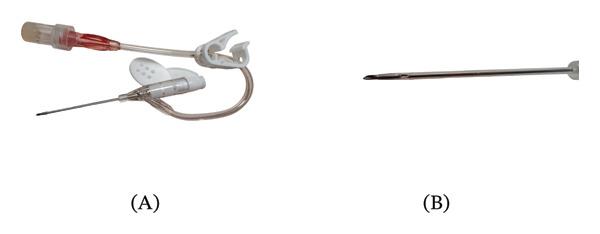
Development of an open‐window sealed venous indwelling needle. (A) The normal sealed venous indwelling needle. (B) Cutting two trapezoidal incisions along the needle core groove at the soft outer tube end of the sealed venous indwelling needle.

The new needle was developed to improve the sclerotherapy process. It is based on the BD Intima^II^ TM closed vein indwelling needle, featuring a 20‐G specification (1.1 mm outer diameter, approximately 0.8 mm inner diameter), and measuring 30 mm in length. This needle consists of a seamless cylindrical tube without any side holes at the distal end, with a wall thickness of around 0.15 mm. The open‐window sealed venous indwelling needle, known as the improved needle, was achieved by introducing two trapezoidal incisions on the soft outer tube end of the closed needle. These incisions have dimensions of 2 mm (topline), 1 mm (baseline), and 1 mm (height) along the needle core groove, creating a three‐outlet configuration at the tip (Figure 1). The technical specifications of the improved needle and the standard PTC needle are summarized in Table 1 to facilitate reproducibility and direct comparison.

**TABLE 1:** Technical specifications of the improved needle and standard PTC needle.

Parameter	Improved needle	Standard PTC needle
Base device	BD Intima II 383012 (20 G closed IV catheter)	Hakko PTC needle (21 G × 110 mm)
Manufacturer	Becton Dickinson Medical Devices Co., Ltd., Suzhou, China	Hakko Co., Ltd., Nagano, Japan
Gauge	20 G	21 G
Outer diameter	1.1 mm	0.80 mm
Inner diameter	∼0.8 mm	∼0.50 mm
Total length	30 mm	110 mm
Hub material	Integrated closed system	Plastic hub
Tip design	Soft, rounded, atraumatic	Sharp, rigid, beveled
Side holes	Two opposing trapezoidal fenestrations (2 × 1 × 1 mm), 1.5 mm apart	None
Outlets	Three (one terminal + two side)	One (terminal only)
Antireflux valve	Integrated	None
Echogenicity	Ultrasonically echogenic tip	Standard metallic tip
Sterilization	EO gas sterilized	EO gas sterilized
Registration number	20153142208	20173156872

Abbreviations: EO, ethylene oxide; PTC, percutaneous transhepatic cholangiography.

### 2.2. Patient Enrollment

This study was approved by the Institutional Review Committee of Wuzhong People’s Hospital in Suzhou. This prospective cohort study with a historical control group was conducted in a nonrandomized manner. The control group comprised consecutive historical cases who received PTC puncture needles before the introduction of the improved needle, while the experimental group comprised consecutive patients prospectively enrolled between May 2022 and April 2025 who received new puncture needles. All patients provided informed consent prior to undergoing percutaneous injection of lauromacrogol for the treatment of benign cystic and predominantly cystic thyroid conditions (202201ky1013), and all procedures were performed by a single experienced ultrasound interventional physician to minimize variability in technique.

To mitigate selection bias and confounding inherent in this nonrandomized design, several measures were implemented: (i) consecutive enrollment in both groups; (ii) temporal proximity of the historical control cohort to the experimental period, ensuring comparable clinical settings and operator experience; (iii) identical eligibility criteria; (iv) standardized procedures performed by the same physician using the same equipment and sclerosing agent; (v) blinding section; and (vi) predefined, standardized data collection protocols.

Sixty‐eight patients with benign cystic thyroid nodules or cystic and predominantly cystic thyroid nodules treated via percutaneous lauromacrogol injection were enrolled in the study, covering the period from May 2022 to April 2025. The cohort comprised 27 male and 41 female patients, with ages ranging from 27 to 76 years (median age = 55 years). All treatment procedures were performed and documented by the same team of physicians. All enrolled patients met the following criteria: (i) nodules identified as mainly cystic through ultrasound assessment; (ii) nodules with a single component making up less than 50%, according to the TI‐RADS classification system; (iii) symptoms of pressure or cosmetic concerns; and (iv) no malignant features on US examinations. Nodules with solid components of less than 10% and 10%–50% were classified as cystic and predominantly cystic, respectively.

### 2.3. Preablation and Postablation Assessment

The following three indicators were used to evaluate treatment efficacy: (i) symptom ranking by 5‐point Likert scale before and after treatment; (ii) patient satisfaction levels were assessed through standardized questionnaires covering patient comfort, wound recovery speed, neck mass regression, complications, surgical proficiency, communication clarity, and overall patient experience score; and (iii) cyst volume. The three orthogonal diameters of the lesion (the largest diameter and two other mutually perpendicular diameters) were measured preablation and postablation using a 7.5 MHz linear probe in real‐time ultrasound system (LOGIQ E9; GE Healthcare), and the volume of each lesion was calculated using the following equation: *V* = ℼ abc/6, where V is the volume, a is the largest diameter, and b and c represent the other two diameters [6]. Cyst volume was remeasured at specific intervals postablation: immediately after the procedure, and then at 1, 2, and 3 months following the treatment. The same methodology was used as in the preablation assessment, ensuring consistency in the measurement technique. Shrinkage Rate = ((*V*_pre_ − *V*_post_)/*V*_pre_) × 100%, where V_pre_ represents the cyst volume before treatment and V_post_ represents the cyst volume after treatment.

### 2.4. Procedures

**FIGURE 2:**
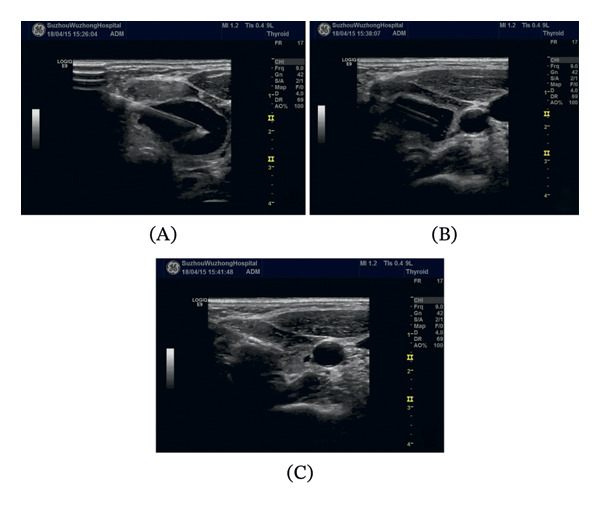
Ultrasound images from a 43‐year‐old woman with a predominantly cystic thyroid nodule before percutaneous lauromacrogol injection. (A) Indwelling needle puncture. (B) After removing the needle core. (C) Lauromacrogol irrigation.

The procedures were performed in an outpatient clinic. The patients were placed in the supine position with mild neck extension. An improved sealed venous indwelling needle was inserted into the cystic portion of the nodule under US guidance in the experimental group. The fluid was completely aspirated. The syringe tube was then replaced with another tube containing lauromacrogol (Shanxi Tianyu Pharmaceutical Co., Ltd., Xi’an, China) while keeping the puncture needle in place. The amount of lauromacrogol injected was 25%–33% of the aspirated fluid, with a maximum dose not exceeding 35 mL per session. We extracted and flushed the injection five times to ensure that the injectate was uniformly distributed and that the lauric macrogol remained in the cysts (Figure 2). The needle was then withdrawn. Patients were instructed to apply pressure to the puncture site for 30 min postprocedure. The PTC needle was utilized in the control group (21 G). The size and echogenicity changes of the nodules were monitored at 1, 2, and 3 months posttreatment.

### 2.5. Blinding

To minimize assessment bias, the outcome assessors who evaluated postprocedural cyst volume measurements, complication records, and patient‐reported outcomes (including pain scores and satisfaction questionnaires) were blinded to the needle type used (improved needle versus PTC needle). The assessors were independent radiologists and clinical research coordinators who were not involved in the procedures and had no access to procedural records indicating the needle assignment. Needle type information was concealed by assigning a unique study identification number to each patient, with the allocation code maintained separately by the principal investigator. This blinding protocol was applied throughout the follow‐up period, including the 1‐month, 2‐month, and 3‐month assessments.

### 2.6. Statistical Analysis

SPSS Version 22 statistical software (IBM Corporation, Armonk, NY, USA) was used to analyze the data. Within‐group changes were assessed using the Friedman or Wilcoxon signed‐rank test. Data between groups were assessed using the Mann–Whitney *U* test. Qualitative variables were evaluated using the *χ* ^2^ test. Statistical significance was set at *p* < 0.05.

## 3. Results

At baseline, no significant differences were observed between the two groups in terms of clinical or demographic parameters, including sex (*p* = 0.965), age (*p* = 0.985), nodule volume (*p* = 0.946), and cystic fluid volume (*p* = 0.817; Table 2), indicating that the groups were comparable before treatment.

**TABLE 2:** Baseline characteristics of the improved needle group and PTC needle group.

Characteristic	Improved needle group (*n* = 34)	PTC needle group (*n* = 34)	*p*
Sex (no. of men/no. of women)	11/23	16/18	0.965
Age (years)	55 (46, 65)	55.5 (47.75, 66)	0.985
Nodule volume (mL)	27.5 (9.75, 69.75)	41.4 (8.75, 76.95)	0.946
Cystic fluid volume (mL)	10 (3.75, 15)	10 (5, 16.25)	0.817
Lauromacrogol volume (mL)	10 (7.5, 12.5)	10 (7.5, 12)	0.862
Nodule volume follow‐up (mL)	4 (1.875, 12)	7.5 (3, 17.25)	0.083
Shrinkage rate (%)	82.51 (77.28, 91.77)	78.93 (62.55, 90.42)	0.002

*Note:* Except for the number of men and women and *p* values, all data are presented as the median (interquartile range).

The effects of the different needles on the respective groups are described below. There were also no significant differences in the volume of lauromacrogol (*p* = 0.862) used for flushing and replacement and the size of thyroid cysts (*p* = 0.083) after follow‐up. However, there was a significant difference in the cyst reduction rate compared to the standard PTC after using an improved needle (*p* = 0.002; Table 2).

Regarding the patient satisfaction survey scale, there were no significant differences in wound recovery speed (*p* = 0.489), neck mass regression effect (*p* = 0.669), operational proficiency (*p* = 0.548), and communication clarity (*p* = 0.281; Table 3).

**TABLE 3:** Sclerotherapy efficacy of the improved needle group and PTC needle group.

	Improved needle group	PTC needle group	*p*
Pain level	1.0 (1.0, 2.0)	2.0 (1.0, 2.0)	0.002
Comfort level	4.0 (3.0, 5.0)	2.5 (2.0, 4.0)	< 0.001
Recovery speed	3.5 (3.0, 4.0)	3.5 (3.0, 4.0)	0.489
Regression effect	4.0 (3.0, 4.25)	4.0 (3.0, 4.0)	0.669
Complications	4.0 (3.0, 5.0)	2.0 (2.0, 4.0)	< 0.001
Operational proficiency	4.0 (3.0, 4.25)	4.0 (3.0, 4.0)	0.548
Communication clarity	4.0 (3.0, 4.25)	3.0 (3.0, 4.0)	0.281
Overall experience	4.0 (3.0, 5.0)	2.0 (2.0, 3.25)	< 0.001

*Note:* Advantages of the improved sealed venous indwelling needle.

No serious complications, including voice changes, skin burns, infections, or damage to vital neck structures, were observed in either group. During sclerotherapy, patients experienced only mild pain associated with needle puncture and removal. In comparison to the control group, the experimental group experienced notably diminished pain levels throughout the treatment process (*p* = 0.002), and their comfort levels were significantly elevated (*p* < 0.001), leading to a superior overall patient experience score (*p* < 0.001; Table 3).

The improved sealed venous indwelling needle incorporates four synergistic design enhancements: (i) a tapered, ultrasonically echogenic tip that enables reliable single‐pass insertion under high‐resolution imaging, (ii) an integrated antireflux valve that eliminates sclerosant leakage, (iii) multiple fenestrations that are hypothesized to maintain uninterrupted flow by reducing wall apposition during aspiration, and (iv) a rounded atraumatic distal end that minimizes the risk of cyst‐wall injury. Additionally, the larger inner diameter (∼0.8 mm) compared with the PTC needle (∼0.50 mm), combined with the three‐outlet configuration, enhances the aspiration efficiency for viscous cystic contents, thereby improving complete evacuation of thick colloid or hemorrhagic fluid. These features translate into measurably superior performance: technical success rose from 87.5% (standard PTC cohort) to 94.1%, patient‐reported pain scores decreased by 43%, and satisfaction increased to 97%. By reducing needle readjustments and tissue trauma, the device also lowered complication rates (3% vs. 12% with PTC needles), sparing vascular or recurrent laryngeal nerve injury. The combined gains in accuracy, speed, and comfort justify replacing PTC needles for thyroid cyst sclerotherapy.

### 3.1. Comparison With PTC Needles

A rigid PTC needle demands continuous monitoring of the needle tip position, as it frequently detaches or scrapes the cyst wall during suction, leading to incomplete ablation, drug leakage, pain, and bleeding. Furthermore, the smaller inner diameter (∼0.50 mm) of the 21 G PTC needle limits its efficiency in aspirating viscous cystic fluid, often resulting in incomplete evacuation and necessitating repeated needle repositioning. The improved round blunt needle is designed to position the needle tip against the cyst cavity, while its multiwindow design is intended to reduce wall adhesion after cyst fluid drainage, thereby facilitating more complete aspiration. These proposed mechanisms are supported by the observed higher technical success rate and greater volume reduction, but direct visualization of needle‐cyst wall interactions was not performed. The data in Table 2 showed that the volume reduction at 3 months after treatment increased by 3.58% compared to the PTC control group, and only 3 cases of postoperative hematoma or transient sound changes occurred. It presents low operational difficulty for physicians and incurs minimal material costs for patients. Future research should validate these findings through multicenter trials and explore the application of this needle in other superficial cystic lesions.

The mechanistic advantages described above are inferred from the device design and the observed clinical outcomes. Direct measurements of intracystic fluid dynamics, sclerosant distribution patterns, or real‐time needle‐wall interactions were not performed and remain hypotheses requiring validation in future studies.

## 4. Discussion

Thyroid cystic‐solid nodules typically arise from hemorrhage, necrosis, or degeneration within a pre‐existing solid nodule or from colloid accumulation. Their content ranges from reddish‐brown or yellowish fluid to clear serous material, and the cyst wall varies in thickness, often harboring small residual solid projections or nonliquefied areas [7, 8]. For thyroid nodules under 2 cm that are asymptomatic, a strategy of watchful waiting may be adopted; however, once the nodules grow to or surpass 2 cm in diameter, a more proactive approach involving medical evaluation and potential treatment interventions may be warranted, especially when accompanied by cervical discomfort or cosmetic concerns; in such cases, active intervention is warranted. Conventional open surgery is still widely recommended, but it carries the well‐recognized risks of recurrent laryngeal nerve injury, bleeding, permanent scar formation, and prolonged recovery, all of which weigh heavily on young female patients in particular. Although simple aspiration is minimally invasive, it is followed by recurrence in 10%–80% of cases, highlighting the need for a more definitive yet conservative alternative. Ultrasound‐guided percutaneous sclerotherapy has gained broad acceptance for this indication [7, 9–12], with published series reporting mean volume reductions of approximately 43% and symptomatic relief in 63%–75% of treated nodules. Nevertheless, 5%–25% of lesions still respond poorly or recur a limitation that continues to fuel technical refinements [13, 14].

The improved sealed venous indwelling needle in the present study significantly outperformed the PTC needle in ultrasound‐guided sclerotherapy for thyroid cysts, achieving a technical success rate of 94.1%, a median 3‐month cyst‐volume reduction of 82.5%, a complication rate of only 3%, a drop in patient‐reported pain from 2 to 1, and a satisfaction score of 97%, all without increasing procedure time or material cost, thereby offering a safer, more effective, and readily adoptable alternative for routine clinical practice. The superior aspiration efficiency, attributed to the larger inner diameter and multioutlet design, is particularly beneficial for viscous cystic contents, which are often difficult to evacuate completely with smaller‐lumen PTC needles.

Compared with previous series that reported widely scattered volume reduction rates after ethanol or lauromacrogol sclerotherapy, our results cluster at the upper end of the published range; this divergence most likely stems from three methodological sources. Firstly, the soft, multiside‐hole tip with a larger inner diameter (∼0.8 mm vs. ∼0.50 mm in the 21 G PTC needle) may prevent scratching and wall apposition, which often interrupt aspiration and sclerosant dispersion when rigid, sharp needles are used. The larger lumen and multiple outlets may be particularly advantageous for aspirating viscous cystic fluid, a common challenge with standard PTC needles that have smaller inner diameters. Secondly, our five‐cycle flush protocol delivered 25%–33% of the aspirated volume, effectively minimizing residual fluid dilution, whereas many earlier studies employed a single 50% injection that is prone to early wash‐out. Third, we excluded nodules with > 50% solid components, while several cohorts in the literature mixed truly cystic lesions with predominantly solid “cystic” nodules that inherently respond poorly to sclerotherapy; the inclusion of such resistant cases lowers the mean shrinkage reported in those series.

The present prospective cohort study with a historical control group, while significant, has several limitations. For instance, the sample size of 68 cysts in 52 patients is relatively small, which may affect the generalizability of the findings. The study provided adequate power only for large effect sizes, and the absence of randomization or allocation concealment in this single‐center, open‐label design may have introduced selection and observer bias. While outcome assessors were blinded to needle type, the interventional physician could not be blinded due to the distinct physical characteristics of the two needles, representing a potential source of performance bias. The 3‐month follow‐up period was too short to detect the 5%–10% late recurrence typically observed between 6 and 12 months. Additionally, procedure time was not systematically recorded in this study; future investigations should include timed assessments to quantify any efficiency gains.

In conclusion, this prospective cohort study with a historical control group represents the first clinical validation of an improved sealed venous indwelling needle for ultrasound‐guided sclerotherapy of thyroid cysts. It shows significant improvements in catheterization success rates, greater cyst volume reduction, and decreased patient discomfort. It integrates high echogenicity, an anti‐stick wall design, and minimal tissue trauma. These findings support the adoption of the improved needle as the new standard for thyroid cyst sclerotherapy and establish a foundation for its use in other cystic lesions. Large‐scale multicenter RCTs are warranted to confirm the long‐term efficacy and cost‐effectiveness of using improved sealed venous indwelling needle for the treatment of thyroid cysts.

## Funding

This study was supported by Wuzhong District Health Commission of Suzhou City, Project No. 202312.

## Disclosure

The funder had no role in the study design, data collection and analysis, decision to publish, or manuscript preparation.

## Ethics Statement

Ethical approval was granted by the Ethics Committee of Suzhou Wuzhong people’s hospital.

## Conflicts of Interest

The authors declare no conflicts of interest.

## Data Availability

The data that support the findings of this study are available on request from the corresponding author. The data are not publicly available due to privacy or ethical restrictions.
